# Nutrient-Dependent Mitochondrial Fission Enhances Osteoblast Function

**DOI:** 10.3390/nu15092222

**Published:** 2023-05-08

**Authors:** Ciro Menale, Giovanna Trinchese, Immacolata Aiello, Giulia Scalia, Monica Dentice, Maria Pina Mollica, Nal Ae Yoon, Sabrina Diano

**Affiliations:** 1 Department of Clinical Medicine and Surgery, University of Naples “Federico II”, 80131 Naples, Italy; imma.aiello@yahoo.it (I.A.); monica.dentice@unina.it (M.D.); 2 Department of Biology, University of Naples “Federico II”, 80126 Naples, Italy; giovanna.trinchese@unina.it (G.T.); mpmollic@unina.it (M.P.M.); 3 CEINGE-Biotecnologie Avanzate Franco Salvatore, 80145 Naples, Italy; scalia@ceinge.unina.it; 4 Centro Servizi Metrologici e Tecnologici Avanzati (CeSMA), Complesso Universitario di Monte Sant’Angelo, 80126 Naples, Italy; 5 Task Force on Microbiome Studies, University of Naples “Federico II”, 80138 Naples, Italy; 6 Institute of Human Nutrition, Columbia University Irving Medical Center, New York, NY 10032, USA; ny2321@cumc.columbia.edu; 7 Department of Molecular Pharmacology and Therapeutics, Columbia University Irving Medical Center, New York, NY 10032, USA; 8 Department of Physiology and Cellular Biophysics, Columbia University Irving Medical Center, New York, NY 10032, USA

**Keywords:** osteoblasts, glucose, palmitate, mitochondrial fission, metabolism, bioenergetics

## Abstract

Background: The bone synthesizing function of osteoblasts (OBs) is a highly demanding energy process that requires nutrients. However, how nutrient availability affects OBs behavior and bone mineralization remain to be fully understood. Methods: MC3T3-E1 cell line and primary OBs (OBs) cultures were treated with physiological levels of glucose (G; 5.5 mM) alone or with the addition of palmitic acid (G+PA) at different concentrations. Mitochondria morphology and activity were evaluated by fluorescence microscopy, qPCR, and oxygen consumption rate (OCR) measurement, and OBs function was assessed by mineralization assay. Results: The addition of non-lipotoxic levels of 25 μM PA to G increased mineralization in OBs. G+25 μM PA exposure reduced mitochondria size in OBs, which was associated with increased activation of dynamin-related protein 1, a mitochondrial fission protein, enhanced mitochondria OCR and ATP production, and increased expression of oxidative phosphorylation genes. Treatment with Mdivi-1, a putative inhibitor of mitochondrial fission, reduced osteogenesis and mitochondrial respiration in OBs. Conclusions: Our results revealed that OBs function was enhanced in the presence of glucose and PA at 25 μM. This was associated with increased OBs mitochondrial respiration and dynamics. These results suggest a role for nutrient availability in bone physiology and pathophysiology.

## 1. Introduction

Bone tissue undergoes continuous remodeling through the coordinated activity of skeletal cells, mainly bone-synthesizing osteoblasts (OBs) and bone-resorbing osteoclasts (OCs) [1,2,3]. Their physiological cellular activities ensure bone growth and maintenance, whereas alterations of the functions of these cells lead to skeletal disorders, characterized by changes in bone mineral density [4]. The homeostatic regulation of bone metabolism is an energy-intensive process that requires a high metabolic demand. Emerging data have highlighted the importance of cellular nutrient utilization and energy generation in OBs. Glucose has been recognized as the fundamental nutrient for the maintenance of bone homeostasis serving as the main energy source for OBs, since these cells and their mesenchymal progenitors primarily metabolize glucose mostly into lactate through glycolysis to meet ATP demand (producing almost 80% of ATP) during the early stages of proliferation and differentiation [5,6,7,8,9,10]. On the other hand, nutrients, such as lipids and amino acids, i.e., glutamine, are required during a later stage of maturation of OBs when matrix production and mineralization processes occur [8,9,10,11]. Specifically, lipids provide between 40 and 80% of the energy provided by glucose in OBs and are metabolized in the mitochondrial matrix through fatty acids β-oxidation (FAO) contributing to mitochondrial ATP production [12]. Fatty acids overload has been shown to be detrimental to OBs function, although the regulation of OBs by physiological levels of lipids is still unclear [9,10,12]. Specifically, palmitic acid (palmitate, PA), a 16-carbon long-chain saturated fatty acid, is the most common and abundant lipid in the human body, produced endogenously in tissues as precursors for several fatty acids, and comes from the diet [13,14]. Although PA has been extensively studied for its lipotoxic effects on OBs in inducing apoptosis, mitophagy, autophagy, and inflammation when present at supraphysiological concentrations [13,15,16,17,18,19], emerging evidence suggests a role for PA in bone physiology, mainly through the modulation of mitochondria metabolism [9,10]. Mitochondria play a pivotal role in cell adaptation to nutrient availability through their ability to alter their dynamics and functions [20]. Mitochondria dynamics, defined as fusion and fission cycling, occurs in response to various metabolic stimuli influencing mitochondrial bioenergetics and controlling cellular calcium homeostasis, redox signaling, and autophagy [21,22]. It has been shown that modification of mitochondrial morphology and activity occur during OBs progenitor cell differentiation and mature OBs mineralization [23,24,25,26]. In support of this, mature OBs show a high number of mitochondria and exhibit active oxidative phosphorylation (OXPHOS) and fatty acid oxidation (FAO), and these processes are greater during collagen deposition and mineralization [9,11,27]. However, how fatty acids availability affects OBs mitochondrial function and dynamics and OBs cellular function remains to be further elucidated. Here we show that in murine calvarial pre-osteoblastic cell line MC3T3-E1 and primary calvarial osteoblasts, the addition of non-lipotoxic PA to glucose increases OBs mitochondrial oxygen consumption rate which was associated with increased mitochondrial fission through activated DRP1 and enhanced OBs mineralization.

## 2. Materials and Methods

### 2.1. Reagents and Chemicals

Palmitic Acid (sodium palmitate, PA), Bovine Serum Albumin (BSA) fatty acid-free, Thiazolyl Blue Tetrazolium Bromide (MTT), Trypan blue solution, Alizarin Red S (ARS), 4% Paraformaldheyde (PFA); Ascorbic acid, b-glycerophosphate, dexamethasone, mitochondrial division inhibitor compound 1 (Mdivi-1), α-minimum essential medium (αMEM) and standard chemicals are purchased from Sigma-Aldrich (St. Louis, MO, USA).

### 2.2. Cell Cultures and Treatments

The murine osteoblastic MC3T3-E1 (clone 14) cell line (ATCC) was cultured in α-minimum essential medium (αMEM) supplemented with 10% fetal bovine serum (FBS, GIBCO Thermo Fisher Scientific, Waltham, MA, USA), 2 mM glutamine and 1% P/S (Sigma-Aldrich) (Basal Medium, BM). Cells were cultured and expanded by incubation at 37 °C and 5% CO_2_ and the medium was replaced every 2 days. Cells were then used for subsequent experiments.

Primary osteoblasts (pOBs) were obtained as described previously [4]. Briefly, calvariae were dissected from C57BL/6J newborn mice (PN3-4), cleaned from adherent soft tissues, and then sequentially digested in 1× HBSS solution (Sigma-Aldrich) containing 1 mg/mL collagenase type IV (Sigma-Aldrich), 0.025% Trypsin (GIBCO Thermo Fisher Scientific) and 1% P/S, at 37 °C. The cells from digestion fractions 2–4 were collected and then plated in a 6-well plate in BM until cells reached confluence. Cells were then used for subsequent experiments up to passage 3. Animals were handled according to national and European Community guidelines, and protocols were approved by the Institutional Animal Care and Use Committee (IACUC) of the University of Naples “Federico II” (protocol n. 354/2019-PR). For subsequent experiments MC3T3-E1 cells were cultured in Dulbecco’s Modified Eagle Medium (DMEM, GIBCO Thermo Fisher Scientific) supplemented with 10% fetal bovine serum (FBS), 2 mM glutamine, and 1% P/S, in the presence of 5.5 mM glucose (resembling in vivo physiological fasting condition) [28] alone or in combination with palmitate (PA). The culture medium with supplementations was replaced every 2 days.

PA was prepared as 8 mM stock solution dissolving PA in NaCl 150 mM at 70 °C and then conjugated with 10% fatty acid free-BSA in NaCl 150 mM (Sigma-Aldrich). The cells were treated with different PA concentrations, as described in specific experiments, supplementing appropriate amounts of the PA/BSA conjugate to the cultured cells. Control cells in normal glucose condition (5.5 mM) were treated with the same amount of BSA without PA conjugation (hereafter referred to as G). PA supplementation to the G conditions is identified with the G+PA [12.5 μM–800 μM] treatment group. For the mitochondrial fission inhibition experiments the Mitochondrial Division Inhibitor 1 (Mdivi-1) compound at 25 μM concentration was added to the G+PA treatment (G+PA+Mdivi-1).

### 2.3. Cell Proliferation and MTT Assay in MC3T3-E1 Cells

MC3T3-E1 cell viability and proliferation were evaluated through the standard dye exclusion method with Trypan blue by seeding 1 × 10^4^ cells in 96 multiwell plates. After 16 h, cells in G conditions were treated for 96 h with PA concentrations ranging from 12.5 μM to 800 μM, according to the literature [15,29,30] for the higher concentrations and using also lower amounts. The culture medium with supplementation was replaced every 2 days. Cells were manually counted to assess PA lipotoxicity. 

Cell proliferation was also evaluated by flow cytometry using the CFSE probe (Invitrogen, Thermo Fisher Scientific, Waltham, MA, USA). MC3T3-E1 cells (1 × 10^6^) were suspended in BM containing CFSE 5 µM. Then cells were incubated for 20 min at 37 °C in the dark. Next, the cells were washed in PBS to remove the excess of the probe, resuspended in BM, and plated in 6-well plates (1.5 × 10^5^ cells/well). After 16 h, cells in G conditions were treated for 96 h with selected PA concentrations ranging from 12.5 μM to 100 μM, excluding cytotoxic PA amounts. The culture medium was replaced every 2 days. Cells were then trypsinized, collected, and resuspended in PBS for further analysis by flow cytometry using FACS Canto II (Becton Dickinson -BD Bioscience, Franklin Lakes, NJ, USA), and the data were analyzed by FlowJo software v.10 (BD Life Sciences). Data are displayed as count normalized to mode and expressed as Mean Fluorescence Intensity (MFI). After excluding cytotoxic PA amounts, also metabolic activity of MC3T3-E1 cells was tested by MTT assay to grossly determine the mitochondrial metabolic activity upon G+PA treatments (ranging from 12.5 μM to 100 μM) for 96 h, following standard procedures, and the culture medium was replaced every 2 days. Results are expressed as metabolic activity relative fold to time 0 (t0) before treatments, over time. On the bases of the observations obtained by these experiments, PA 25 μM was selected as the non-cytotoxic and metabolically relevant PA dose for subsequent analyses (hereafter, referred to as G+PA25). 

### 2.4. Osteogenic Differentiation 

MC3T3-E1 cells and pOBs were plated in 24-well plates in BM and when cells reached confluence were cultured in osteogenic medium in DMEM, as above, supplemented with 50 µM ascorbic acid, 10 mM β-glycerophosphate and 100 nM dexamethasone (Sigma-Aldrich), for 14 days (for the MC3T3-E1 cells) or 7 days (for pOBs). Cells were induced to mineralize in G or G+PA25 or G+PA25+Mdivi-1 treatment conditions. The culture medium with supplementation was replaced every 2 days.

Cells were fixed for Alkaline Phosphatase staining with the Leukocyte Alkaline Phosphatase kit (Sigma-Aldrich) following the manufacturer’s instruction, or for the evaluation of calcium mineralization by Alizarin Red S (ARS) staining and quantification according to standard procedures, reading absorbance at λ405 nm [4]. ARS data are represented as fold change relative to G condition. ALP+ and ARS stained OBs were photographed using an inverted microscope Leica DMi1 (Leica Microsystems, Wetzlar, Germany). Alkaline Phosphatase activity (ALP activity) was also evaluated by the Alkaline Phosphatase Activity Assay Kit (Elabscience, Houston, TX, USA), according to the manufacturer’s protocol and calculated as enzymatic units/mg protein. 

### 2.5. Gene Expression Analysis

Total RNA was extracted from cell cultures using the PureZOL™ Reagent (Bio-Rad, Hercules, CA, USA), following the manufacturer’s instructions. Reverse transcription was carried out using 1.0 μg total RNA and High Capacity cDNA Reverse Transcription Kit (Applied Biosystems, Foster City, CA, USA). qPCR was performed using iTaq Universal SYBR Green Supermix (Bio-Rad) and gene-specific primers as detailed in Supplementary Table S1. The amplification was performed using the CFX Connect Real-Time PCR Detection System (Bio-Rad) with the following cycling conditions: cDNA denaturation and polymerase activation step at 95 °C for 3 min followed by 40 cycles of denaturation at 95 °C for 15 s and annealing at 60 °C for 30 s; melting curve analysis step at 65 °C to 95 °C with 0.5 °C increment for 5 s/step. The relative gene expression analysis of target genes was conducted in comparison with the 18 s housekeeping control gene following the comparative 2^−ΔCt^ method. The normalized expression was calculated as fold change mRNA level versus G control condition.

For mitochondrial DNA (mtDNA) copy number quantification, total DNA was obtained from cell culture samples using standard procedures, and 30 ng total DNA was used as a template. The mitochondrial mtND-1 gene amplification levels were normalized against the nuclear RNAase P gene as previously described [31].

### 2.6. Mitochondrial Morphology, Membrane Potential, and ROS Production Analysis

Mitochondrial morphology and mitochondrial membrane potential were evaluated using the MitoTracker Red CMXRos dye (Invitrogen, Thermo Fisher Scientific, Waltham, MA, USA), according to the manufacturer’s instructions. MitoTracker Red CMXRos fluorescent dye labels mitochondria in live cells and its accumulation within mitochondria is dependent upon membrane potential. Briefly, glass-cultured osteogenic-induced MC3T3-E1 cells and pOBs were incubated in PBS containing 500 nM of the probe in the dark at 37 °C for 30 min. The cells were then washed twice in PBS, fixed in 4% PFA and nuclei were stained with DAPI 1 μg/mL (Sigma Aldrich, St. Louis, MO, USA). Images were acquired with fluorescence microscope LEICA DMi8 equipped with Leica Application Suite LAS X Imaging Software. ImageJ software (NIH Image, Bethesda, MD, USA) was used to analyze mitochondrial morphology (through the Mitochondria Analyzer Plug-in and shape descriptors), and MitoTracker Red CMXRos fluoresce intensity as a measure of mitochondrial membrane potential (Δψm), represented as fold fluorescence intensity/cell area versus G condition. MitoTracker Red CMXRos fluorescence in MC3T3-E1 cells and pOBs were also analyzed by flow cytometry. Briefly, stained cells were suspended in PBS/2% FBS/5 mM EDTA, and then analyzed using a FACSCanto II flow cytometer (BD Bioscience) to detect probe Mean Fluorescence Intensity (MFI). Data were analyzed using FlowJo Software and displayed as count normalized to mode. 

Mitochondrial reactive oxygen species production (mtROS) was evaluated using the MitoSOX Red Mitochondrial Superoxide Indicator probe (Invitrogen Thermo Fisher Scientific), according to the manufacturer’s instructions. MitoSOX Red Mitochondrial Superoxide Indicator fluorogenic dye is specific for mitochondria in live cells and its oxidation by superoxide generates red fluorescence. Briefly, glass-cultured osteogenic-induced MC3T3-E1 cells and pOBs were incubated in PBS containing 5 mM of the probe in the dark at 37 °C for 20 min. The cells were then washed twice in PBS, fixed in 4% PFA and nuclei were stained with DAPI 1 μg/mL (Sigma Aldrich). Images were acquired as above. ImageJ software was used to analyze probe fluoresce intensity to evaluate mtROS production, represented as fold fluorescence intensity/cell area versus G condition. 

### 2.7. Immunofluorescence Microscopy Analysis

For immunofluorescence analysis, pOBs were induced to mineralization and treated on glass in the conditions described above. Then, cells were fixed in 4% PFA for 15 min at RT, permeabilized with 0.2% Triton™ X-100 (Sigma-Aldrich) in PBS, and blocked with PBS 5% FBS for 1 h at RT. The primary antibodies anti-p-DRP1[Ser616] (Cell Signaling Technologies, Danvers, MA, USA) were diluted 1:250 in blocking buffer and incubated for 2 h at RT. The secondary antibody conjugated with Alexa Fluor 488 (donkey anti-rabbit IgG, Invitrogen Thermo Fisher Scientific) was diluted in blocking buffer 1:300 at a concentration of 0.0067 mg/mL, and incubated for 45 min at RT. Nuclei were visualized with DAPI (Sigma Aldrich). Images were acquired with fluorescence microscope LEICA DMi8 equipped with Leica Application Suite LAS X Imaging Software. ImageJ software (NIH Image, Bethesda, MD, USA) was used to analyze fluorescence intensity and data were represented as fluorescence intensity/cell area versus G condition.

### 2.8. Western Blot Protein Analysis

Total protein extracts from pOBs, gel electrophoresis, transfer, and visualization were performed using standard Western blotting techniques. Briefly, pOBs cells were homogenized in homemade radioimmunoprecipitation assay (RIPA) buffer supplemented with protease inhibitor (Sigma-Aldrich) and phosphatase inhibitors (1 mM NaF, 2.5 mM, Na_4_P_2_O_7_, 1 mM Na_3_VO_4_, 1 mM β-Glycerophosphate). Protein concentration was assessed using DC Protein Assay Kit II (Bio-Rad). Twenty-five micrograms (25 μg) of protein extracts were separated on a 10% SDS-PAGE, transferred to a nitrocellulose membrane, and probed with an antibody for p-DRP1[Ser616] (Cell Signaling), total DRP1 (BD Bioscence—final concentration 0.25 μg/mL) both diluted 1:1000 in 5% BSA in 20 mM Tris-buffered saline, pH 7.6, 0.1% Tween 20 (TBST), and with an antibody for β-actin (Cell Signaling) diluted 1:1000 in 5% BSA in TBST, washed, and probed with a secondary antibody conjugated with horseradish peroxidase (Bio-Rad) diluted 1:3000 in TBST, and developed using the Immobilon™ Western kit (Millipore, Watford, UK). Images were captured using the ChemiDoc™ MP Imaging System equipped with Image Lab™ Software (Bio-Rad). Band intensity analysis was performed using ImageJ Software.

### 2.9. Seahorse Analysis

Cellular oxygen consumption measurements in MC3T3-E1 cells and pOBs in the different culture conditions were conducted using the Seahorse XFp analyzer and the Cell Mito Stress Test kit (Agilent Seahorse Biosciences, North Billerica, MA, USA). Both MC3T3-E1 cells and pOBs were seeded in a Seahorse mini plate at density 3 × 10^4^ cells/well in BM for 16 h and then confluent cells underwent osteogenic induction in G, G+PA25, and G+PA25+Mdivi-1 conditions for 4 days. Before analyses, the medium was replaced with a Seahorse XF buffered base medium (Agilent, Santa Clara, CA, USA) supplemented with 2 mM glutamine, 1 mM pyruvate, and 5.5 mM glucose at pH 7.4 and equilibrated at 37 °C in a CO_2_ free incubator for 1 h. Basal respiration was evaluated in the presence of the incubation medium alone. Inhibition of mitochondrial ATP production was induced by 1 µM oligomycin, as an inhibitor of the F0-F1 ATPase. Afterward, the mitochondrial electron transport chain was stimulated maximally by the addition of the uncoupler FCCP (2 µM). Finally, inhibitors of complexes I and III were added: rotenone A (0.5 µM) and antimycin (0.5 µM) as detailed previously [32]. Data were collected and analyzed by using the Seahorse Wave Desktop Software. The data normalization of oxygen consumption measurements was performed using the cell number at the end of the experiments, then mitochondrial respiration was expressed as oxygen consumption rate (OCR, pmol/min/cells).

### 2.10. Statistical Analysis

Statistical analysis was performed using Mann–Whitney test or *t*-test when comparing two groups. One-way or two-way ANOVA with Tukey’s post-test was used for multiple comparisons (GraphPad Prism 6.0; GraphPad Software, Inc., La Jolla, CA, USA). Statistical significance was considered when *p* < 0.05 (* *p* < 0.05, ** *p* < 0 .01, *** *p* < 0.001). All data are described in the results as mean ± SD and presented as mean ± SEM in histograms or as box plots showing the median and SD, as indicated.

## 3. Results

### 3.1. PA Effects on Cell Viability and Osteogenesis in MC3T3-E1 Cells

**Figure 1:**
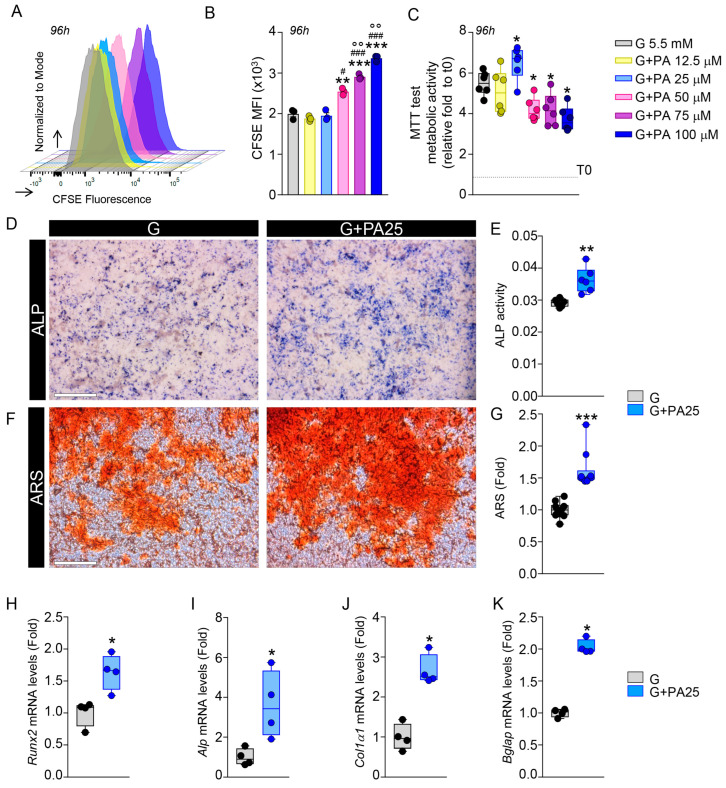
Effect of PA on MC3T3-E1 cell proliferation, metabolic activity, and osteogenesis. MC3T3-E1 cells were treated for 4 days with Glucose 5.5 mM (**G**) plus different PA concentrations ranging from 12.5 μM to 100 μM. (**A**) CFSE representative fluorescence histograms and (**B**) mean fluorescence intensity (MFI) at 96 h after treatments (*n =* 3 per group). (**C**) MTT assay metabolic activity relative fold to t0 at 96 h after treatments (*n* = 6 per group). Osteogenic-induced cells treated with G or G+PA25. (**D**) Representative images for ALP staining (Scale bar 400 μm) and (**E**) ALP enzyme activity (*n* = 6 per group). (**F**) Representative images for ARS staining (Scale bar 400 μm) and (**G**) relative ARS quantization (*n* = 10 per group). (**H**–**K**) qPCR analysis for osteogenic marker genes (*n* = 4 per group). * *p* < 0.05, ** *p* < 0.01, *** *p* < 0.001 vs. G 5.5 mM; # *p* < 0.05, ### *p* < 0.001 vs. G+PA12.5 μM; °° *p* < 0.01 vs. G+PA25 μM.

PA is known to induce a negative effect on OBs viability and proliferation when administered in vitro at supraphysiological concentrations [13,15,19]. In order to examine the cellular and metabolic effects of glucose and PA on OBs, we first evaluated MC3T3-E1 cell viability after incubation of cells with G 5.5 mM (G) plus different concentrations of PA ranging from 12.5 µM to 800 µM to define a PA amount that did not affect cell viability, in order to better study metabolic changes (Supplementary Figure S1A). After 96 h of treatments, we detected a significantly reduced cell number starting from G+PA 50 µM to G+PA 75 µM treatments (cell number, G: 10.5 × 10^5^ ± 2.12; G+PA12.5: 9.5 × 10^5^ ± 0.7; G+PA25: 9.2 × 10^5^ ± 0.42; G+PA50: 6.3 × 10^5^ ± 0.28; G+PA75: 4.25 × 10^5^ ± 0.07). While the G+PA 100 µM to 800 µM treatments dramatically reduced cell number as compared to G treatment alone (cell number, G+PA100: 3.75 × 10^5^ ± 0.35; G+PA200: 1.9 × 10^5^ ± 0.14; G+PA400: 1.35 × 10^5^ ± 0.21; G+PA800: 0.9 × 10^5^ ± 0.14), representing the higher cytotoxic conditions (Supplementary Figure S1A). After excluding the high supraphysiological and cytotoxic PA doses, we evaluated cell proliferation upon treatments of cells with G+PA ranging from 12.5 µM to 100 µM for 96 h, by examining the CFSE probe dilution. As shown in Figure 1A,B, no differences were found in probe fluorescence intensity in G+PA12.5 µM and G+PA25 µM as compared to G treatment alone, also showing a high dilution of the probe (CFSE MFI (×10^3^), G: 1.99 ± 0.12; G+PA12.5: 1.87 ± 0.06; G+PA25: 1.948 ± 0.12). When cells were treated with G+PA50 µM to 100 µM, the retention of the CFSE probe and its fluorescence intensity was higher than G control and as compared to the lower PA doses treatments (CFSE MFI (×10^3^), G+PA50: 2.54 ± 0.08; G+PA75: 2.9 ± 0.06; G+PA100: 3.36 ± 0.07), indicating a lower proliferation rate in cells undergone G+PA50 µM to100 µM as compared to G control and as compared to cells stimulated with lower PA concentrations, indicating a lower ability to dilute the probe according to the limited population doubling. 

We then grossly evaluated cell metabolic activity by MTT test upon treatments of cells with G+PA ranging from 12.5 μM to 100 μM for 96 h (Supplementary Table S2, Supplementary Figure S1B). At the end of the treatments, G+PA doses of 50 µM to 100 µM showed a lower metabolic activity as compared to G alone (Figure 1C, fold at 4 days vs. t0; G: 5.48 ± 0.49; G+PA50: 4.18 ± 0.52; G+PA75: 4.2 ± 0.71; G+PA100: 3.7 ± 0.56), while no differences were observed after G+PA12.5 µM administration. G+PA25 µM, although did not show to have any effect on cell proliferation, showed an increased metabolic activity as compared to G alone, starting at 48 h after treatment, reaching a 6.49 ± 0.8 fold increase vs. t0 after 96 h as compared to G condition (fold vs. t0 G: 5.48 ± 0.49) (Figure 1C and Supplementary Table S2), indicating that PA 25 µM (hereafter, PA25) can be considered as the PA dose that, when added to glucose, does not affect cell viability but possibly favors cell metabolism. To assess whether the observed enhanced metabolic activity could influence cell functionality, we performed an osteogenic differentiation of MC3T3-E1 cells in the G and G+PA25 conditions. As reported in Figure 1D, ALP staining was higher in G+PA25 as compared to G treatment, as well as ALP activity was increased (Figure 1E ALP activity G: 0.0291 ± 0.001; G+PA: 0.036 ± 0.004). Alizarin red staining for the mineralization and its quantization also indicated increased osteogenesis in G+PA25 treatment (Figure 1F,G; G+PA25: 1.61 ± 0.28 ARS fold vs. G). Gene expression analysis of osteogenic markers showed that G+PA25 administration increased mRNA levels of *Runx2* (G+PA25: 1.58 ± 0.37 fold vs. G), *Alp* (G+PA25: 3.62±1.68 fold vs. G), *Col1α1* (G+PA25: 2.66 ± 0.38 fold vs. G), and *Bglap* (G+PA25: 2.03 ± 0.11 fold vs. G) as compared to G condition (Figure 1H–K), confirming that the administration of PA 25 µM to glucose enhanced osteogenesis, possibly promoting cell metabolism.

### 3.2. PA 25 μM Dose Induced Mitochondria Morphological Changes in MC3T3-E1 Cells

**Figure 2:**
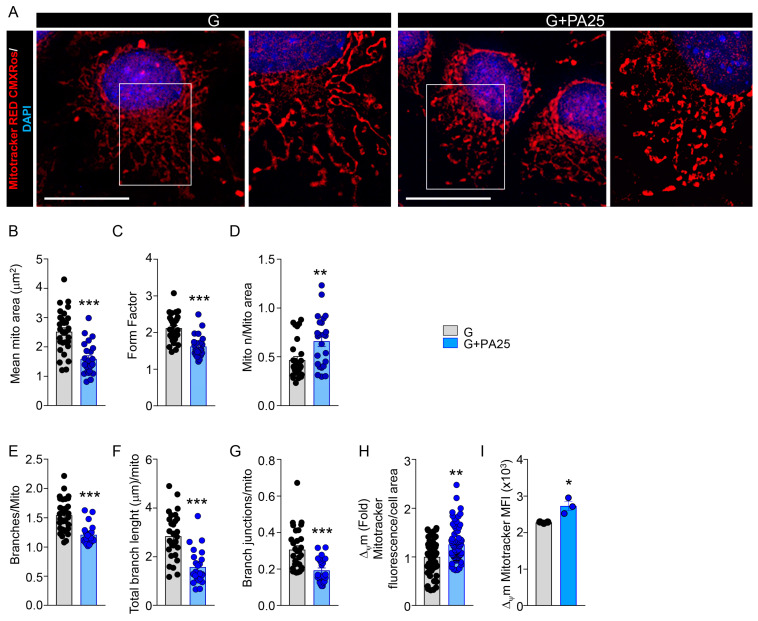
PA 25 μM dose effect on mitochondria morphology in MC3T3-E1 cells. MC3T3-E1 cells undergoing osteogenesis and treated with G or G+PA25 were labeled with Mitotracker Red CMXRos probe. (**A**) Representative images for Mitotracker Red CMXRos staining (scale bar: 25 μm for lower magnification, 40 μm for higher magnification). (**B**–**D**) Morphological parameters evaluation and mitochondrial number/mitochondrial area ratio analysis. (**E**–**G**) Mitochondrial network analysis (three samples per group; *n* = 30 cells for G and *n* = 24 cells for G+PA. (**H**) Δψm (fold) analysis calculated as probe fluorescence intensity/cell area (three samples per group; *n* = 75 cells for G and *n* = 70 cells for G+PA25). (**I**) Δψm analysis represented as Mean Fluorescence Intensity obtained by flow cytometry (*n* = 3 per group). * *p* < 0.05, ** *p* < 0.01, *** *p* < 0.001.

The energy metabolism of the cell is mainly regulated by mitochondrial machinery, and mitochondria morphology adaptation to nutrients plays a pivotal role in regulating energy production and the metabolic fate of the cells [20]. We explored whether the G+PA25 condition was able to influence mitochondria morphology. MitoTracker Red CMXRos staining of osteogenesis-induced MC3T3-E1 cells (Figure 2A) allowed us to evaluate mitochondria morphology through the Mitochondrial Analyzer Plug-in for ImageJ and shape descriptors. Upon G+PA25 administration we observed a reduction of the mitochondria size (Figure 2B; mean area, G: 2.51 μm^2^ ± 0.72, G+PA25: 1.58 μm^2^ ± 0.53) and of the form factor, which describes the putative shape of the mitochondrial object, (Figure 2C; form factor, G: 2.18 ± 0.36, G+PA25: 1.61 ± 0.3) as compared to G alone, indicating smaller and more rounded mitochondria. The mitochondria number over the mitochondria area ratio [33] was increased in G+PA25 treatment as compared to G control (Figure 2D; G: 0.42 ± 0.14, G+PA25: 0.71 ± 0.23), allowing us to hypothesize that a mitochondria fission process might be induced upon PA 25 μM dose administration to G. The mitochondria network analysis showed that the number of mitochondrial branches per mitochondrion, their total length per mitochondrion and their junction number per mitochondrion were decreased in G+PA25 (Figure 2E–G; branches/mito, G: 1.54 ± 0.28, G+PA25: 1.21 ± 0.16; total branches length/mito, G: 2.8 μm ± 0.28, G+PA25: 1.57 μm ± 0.71; branch junctions/mito, G: 0.3 ± 0.11, G+PA25: 0.19 ± 0.06), indicating that PA 25 μM supplementation to glucose resulted in a more fragmented mitochondrial network. Of note, mitochondrial mass and biogenesis were not affected since mtDNA copy number and the mRNA levels of the *Pgc1α* gene did not change after G+PA25 incubation (Supplementary Figure S2A,B). Interestingly, the evaluation of MitoTracker Red CMXRos probe intensity showed a slight but significant increase in mitochondria membrane potential (Δψm) in G+PA25 vs. G alone (Figure 2H, image analysis G+PA25: 1.25 ± 0.36 fold vs. G; Figure 2I, flow cytometry analysis G: 2280 ± 19.08 MFI, G+PA25: 2730 ± 215 MFI. Supplementary Figure S2C displays the correspondent flow cytometry histograms), suggesting an enhancement of mitochondrial metabolic activity.

### 3.3. Mitochondrial Activity Is Enhanced by PA 25 μM in MC3T3-E1 Cells

**Figure 3:**
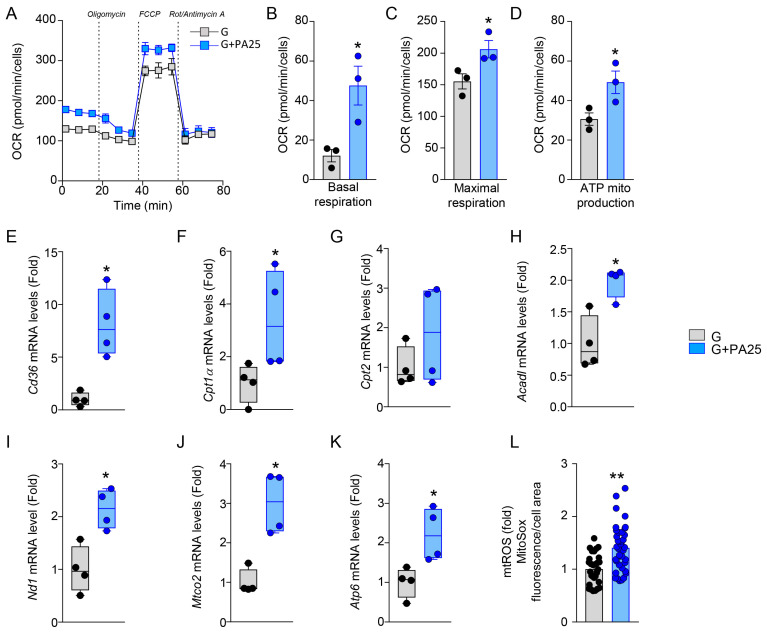
Mitochondrial activity profile upon PA 25 μM administration in MC3T3-E1 cells. (**A**) Seahorse analysis on osteogenic-induced MC3T3-E1 cells undergoing G and G+PA25 treatments, and (**B**–**D**) evaluation of respiratory parameters expressed as OCR, pmol/min/cells (*n* = 3 per group). (**E**–**K**) qPCR analysis of gene expression related to fatty acid metabolism (*n* = 4 per group). (**L**) mtROS production analysis calculated as MitoSox RED probe fold fluorescence intensity/cell area (three samples per group; *n* = 29 cells for G and *n* = 38 cells for G+PA25). * *p* < 0.05, ** *p* < 0.01.

To further elucidate the previous findings, we performed the oxygen consumption rate (OCR, pmol/min/cells) analysis through a palmitate oxidation stress test (Figure 3A). The addition of PA25 to G increased basal respiration (Figure 3B; G: 12.1 ± 5.3 OCR, G+PA25: 47.57 ± 16.9 OCR), enhanced maximal respiration (Figure 3C; G: 155.2 ± 20.7 OCR, G+PA25: 206.2 ± 24.02 OCR), and showed a higher mitochondria-linked ATP production (Figure 3D; *G: 30.6 ± 5.4 OCR*, *G+PA: 49.23 ± 9.8 OCR*) as compared to G alone. These data allowed us to hypothesize that increased mitochondrial respiration and metabolism are induced by administering PA 25 μM to G. To deepen these findings, expression analysis of fatty acids oxidation-related genes was performed. The G+PA25 group showed an increased mRNA level of the fatty acid transporter *Cd36* gene (Figure 3E; G+PA25: 8.15 ± 3.2 fold vs. G) and of the carnitine palmitoyltransferase I (*Cpt1α*) gene (Figure 3F; G+PA25: 3.38 ± 1.9 fold vs. G). No significant differences were detected for the Carnitine Palmitoyltransferase 2 (*Cpt2*) gene (Figure 3G). Furthermore, the expression of *Acadl*, the gene encoding for Acyl-CoA Dehydrogenase Long Chain (a key enzyme in the mitochondrial β-oxidation of fatty acids), resulted increased upon PA administration to G (Figure 3H; G+PA25: 1.95 ± 0.29 fold vs. G). These observations suggested that fatty acid oxidation (FAO) and utilization possibly enhanced OXPHOS. Indeed, *Nd1* (encoding for the mitochondrially encoded NADH:Ubiquinone oxidoreductase core Subunit 1), *Mtco2* (Mitochondrially Encoded Cytochrome C Oxidase II) and *Atp6* (mitochondrially encoded ATP Synthase Membrane Subunit 6) gene expression were elevated in the presence of both energy substrates (Figure 3I–K; *Nd1*, G+PA25: 2.04 ± 0.53; *Mtco2*, G+PA25: 3 ± 0.77; *Atp6*, G+PA25: 2.21 ± 0.66 fold vs. G). Indeed, we also observed that the mitochondrial ROS production (mtROS) was significantly increased in G+PA25 treatments as compared to G alone (Figure 3L, G+PA25: 1.406 ± 0.43 fold vs. G).

### 3.4. Effects of PA 25 μM on Mineralization in Primary OBs

**Figure 4:**
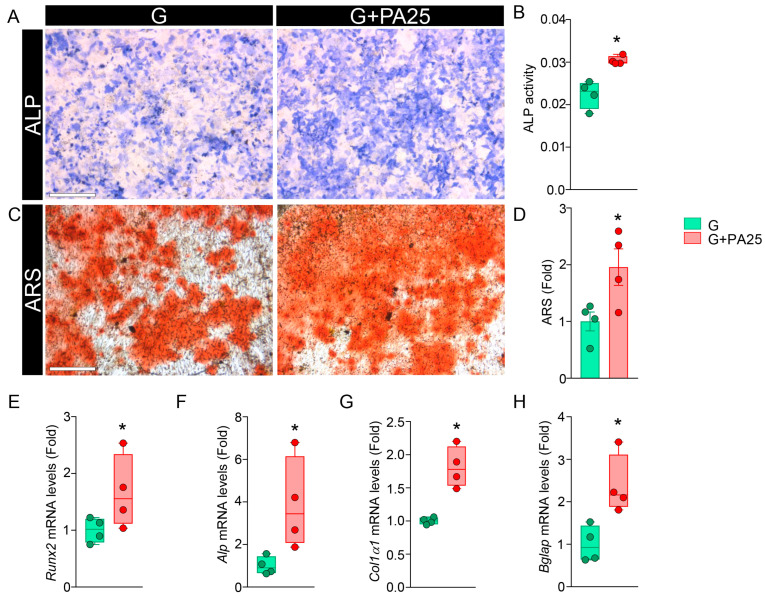
PA 25 μM dose effect on mineralization in pOBs. pOBs in osteogenic conditions treated with G or G+PA25. (**A**) Representative images for ALP staining (Scale bar 400 μm) and (**B**) ALP enzyme activity (*n* = 4 per group). (**C**) Representative images for ARS staining (Scale bar 400 μm) and (**D**) relative ARS quantization (*n* = 4 per group). (**E**–**H**) qPCR analysis for osteogenic marker genes (*n* = 4 per group). * *p* < 0.05.

To deepen PA25’s contribution to metabolic changes in a more physiologically relevant model of OBs, we performed osteogenesis in primary calvarial OBs (pOBs) derived from C57Bl6/J mice, in G and G+PA25 conditions. We confirmed previous results obtained studying the MC3T3-E1 cell line, showing that pOBs undergoing mineralization increased ALP staining in culture (Figure 4A) and ALP activity (Figure 4B; ALP activity G: 0.022 ± 0.003; G+PA: 0.03 ± 0.001) as compared to G alone. The increased osteogenic capacity was corroborated by the enhanced mineralization of pOBs in the G+PA25 conditions as compared to G control, as reported in Figure 4C,D by ARS staining and its quantification (ARS, G+PA25: 1.95 ± 0.64 fold vs. G). Moreover, the gene expression analysis for osteogenic markers confirmed our observations. G+PA25 administration to osteogenic-induced pOBs increased mRNA levels of *Runx2* (G+PA25: 1.67 ± 0.64 fold vs. G), *Alp* (G+PA25: 3.88 ± 2.16 fold vs. G), *Col1α1* (G+PA25: 1.81 ± 0.3 fold vs. G), and *Bglap* (G+PA25: 2.38 ± 0.7 fold vs. G) as compared to the G control condition (Figure 4E–H), confirming that the addition of PA 25 µM to glucose favored mineralization, possibly acting on OBs metabolism.

### 3.5. Mitochondrial Fission Process Is Induced by PA 25 μM Doses in pOBs

**Figure 5:**
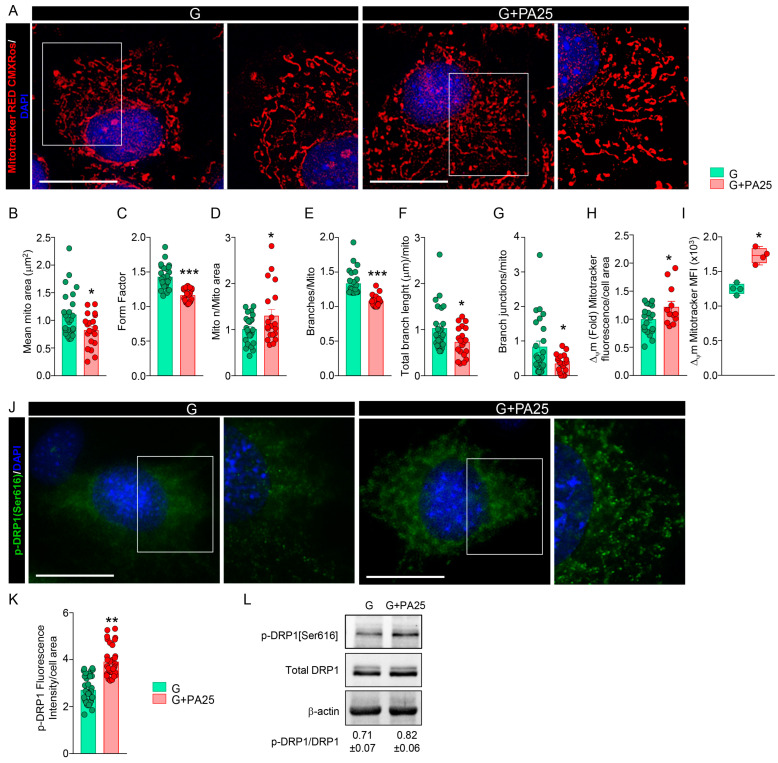
Mitochondrial fission induced by PA25 dose in pOBs. Osteogenic-induced pOBs treated with G or G+PA25 were labeled with a Mitotracker Red CMXRos probe. (**A**) Representative images for Mitotracker Red CMXRos staining (scale bar: 25 μm for lower magnification, 40 μm for higher magnification). (**B**–**D**) Morphological parameters evaluation and mitochondrial number/mitochondrial area ratio analysis. (**E**–**G**) Mitochondrial network analysis (four mice per group; *n* = 25 cells for G and *n* = 20 cells for G+PA. (**H**) Δψm analysis calculated as probe fold fluorescence intensity/cell area (four mice per group; *n* = 20 cells for G and *n* = 12 cells for G+PA25). (**I**) Δψm analysis represented as Mean Fluorescence Intensity obtained by flow cytometry (*n* = 4 per group). (**J**) Representative images for pDRP1[Ser616] immunofluorescence staining (scale bar: 25 μm for lower magnification, 40 μm for higher magnification) and (**K**) relative quantification (four mice per group; *n* = 39 cells for G and *n* = 35 cells for G+PA25) expressed as fluorescence intensity/cell area. (**L**) Representative western blot image and analysis of DRP1 expression in its phosphorylated form related to total DRP1 protein (normalized on actin) (*n* = 3 per group). * *p* < 0.05, ** *p* < 0.01, *** *p* < 0.001.

To investigate mitochondria dynamics changes in the presence of G+PA25 osteogenic culture conditions, pOBs were examined to evaluate mitochondria morphology by MitoTracker Red CMXRos staining (Figure 5A). Upon G+PA25 administration to pOBs we detected a reduction of the mitochondria size (Figure 5B; mean area, G: 1.1 μm^2^ ± 0.41, G+PA25: 0.8 μm^2^ ± 0.29) and of the form factor (Figure 5C; G: 1.43 ± 0.17, G+PA25: 1.16 ± 0.09) as compared to G alone, confirming also in primary cells that mitochondria were smaller and more rounded in shape. The mitochondria number/mitochondria area was also increased in G+PA25 treatment as compared to the G control condition (Figure 5D; G: 1.01 ± 0.3, G+PA25: 1.3 ± 0.61), enforcing our hypothesis of the induction of mitochondria fission process by PA 25 μM dose administration to G. Furthermore, a more fragmented mitochondria network was observed in G condition plus PA 25 μM as compared to G alone, since the number of mitochondrial branches per mitochondrion, their total length per mitochondrion and their junctions number per mitochondrion were decreased in G+PA25 (Figure 5E–G; branches/mito, G: 1.33 ± 0.18, G+PA25: 1.08 ± 0.07; total branches length/mito, G: 1.03 mm ± 0.48, G+PA25: 0.73 mm ± 0.32; branch junctions/mito, G: 0.83 ± 0.8, G+PA25: 0.35 ± 0.26). Of note, also in these settings, no differences were detected in terms of mitochondria mass and biogenesis, as shown in Supplementary Figure S2D,E, where both mtDNA copy number and mRNA levels of *Pgc1α* did not change upon fatty acid stimulation. The analysis of mitochondria membrane potential (Δψm) showed an increased probe fluorescence intensity in G+PA25 vs. G control condition (Figure 5H, image analysis G+PA25: 1.22 ± 0.34 fold vs. G; Figure 5I, flow cytometry analysis G: 1250 ± 76.84 MFI, G+PA25: 1729 ± 111.4 MFI; See also Supplementary Figure S2F displaying correspondent flow cytometry histograms), corroborating the hypothesis of an enhancement of mitochondrial metabolic activity. To finally assess fission process induction, we performed a protein analysis on the master regulator factor of this process, the DRP1 protein. Its active phosphorylated form (pDRP-1[Ser616]) promotes mitochondrial fission, and it was highly expressed in pOBs undergoing G+PA25 treatments as compared to G alone, as it has been demonstrated by immunofluorescence analysis (Figure 5J, K; fluorescence intensity/cell area, G: 2.69 ± 0.55; G+PA25: 3.9 ± 0.63). Moreover, protein analysis of pDRP1[Ser616] in relation to its total form (tot DRP1) confirmed a trend of previous results as shown by western blot evaluation (Figure 5L; densitometric analysis of p-DRP1/tot Drp1 normalized on β-Actin, G: 0.71 ± 0.07; G+PA25: 0.82 ± 0.06). Altogether, these results indicated that in osteogenic-induced pOBs, the administration of PA 25 μM to G induced mitochondria fission as a response to nutrients stimulation.

### 3.6. PA 25 μM Dose Boosts Mitochondrial Oxidative Metabolism in pOBs

**Figure 6:**
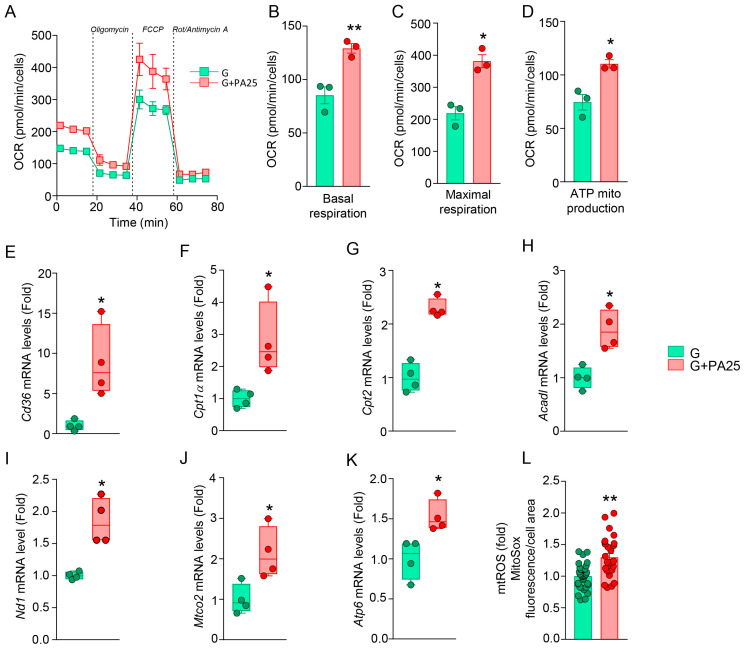
PA 25 μM dose influence mitochondrial metabolic profile in pOBs. Mitochondrial activity profile upon low PA levels administration to G in pOB cells. (**A**) Seahorse analysis on osteogenic-induced pOBs undergoing G and G+PA25 treatments, and (**B**–**D**) evaluation of respiratory parameters expressed as OCR, pmol/min/cells (*n* = 3 per group). (**E**–**K**) qPCR analysis of gene expression related to fatty acid metabolism (*n* = 4 per group). (**L**) mtROS production analysis calculated as MitoSox RED probe fold fluorescence intensity/cell area (four mice per group; *n* = 39 cells for G and *n* = 27 cells for G+PA25). * *p* < 0.05, ** *p* < 0.01.

To investigate mitochondrial function in response to the addition of PA25 to G, pOBs were subjected to measurements of mitochondrial oxidative capacities through OCR evaluation during a palmitate oxidation stress test (Figure 6A). The G+PA25 condition significantly increased basal respiration (Figure 6B; G: 85.27 ± 13.66 OCR, G+PA25: 129.1 ± 7.6 OCR), and maximal respiration (Figure 6C; G: 218.9 ± 35.61 OCR, G+PA25: 381.4 ± 35.2 OCR) as compared to G alone. Mitochondria-linked ATP production was also significantly elevated in G+PA25 (Figure 6D; G: 74.4 ± 12.65 OCR, G+PA: 110.3 ± 6.64 OCR). Altogether, these results confirmed that also in physiologically relevant pOBs the addition of a non-cytotoxic dose of PA to glucose boosted mitochondrial respiration and oxygen consumption to produce energy as ATP. To investigate the influence of G+PA25 treatments on the fatty acids metabolic pathway, an expression analysis of fatty acid metabolism-related genes was performed. The G+PA25 group showed an increased level of the *Cd36* mRNA levels (Figure 6E; G+PA25: 8.8 ± 4.5 fold vs. G), of *Cpt1α* (Figure 6F; G+PA25: 2.81 ± 1.15 fold vs. G) and *Cpt2* (Figure 6G; G+PA25: 2.29 ± 0.17 fold vs. G) as compared to G treatment alone. Indeed, the expression of *Acadl* resulted elevated upon PA 25 μM administration to G (Figure 6H; G+PA25: 1.9 ± 0.36 fold vs. G). These results suggest that the addition of non-lipotoxic levels of PA (25 μM) to glucose may induce FAO and PA utilization by the mitochondria to possibly fuel OXPHOS. In agreement, when both fuels are present, the expression of OXPHOS-related genes resulted increased as compared to G administration (Figure 6I–K; *Nd1*, G+PA25: 1.84 ± 0.059; *Mtco2*, G+PA25: 2.13 ± 0.63; *Atp6*, G+PA25: 1.5 ± 0.19 fold vs. G). To test whether this augmented mitochondrial respiration activity was due to increased OXPHOS and electron transport chain activity, we analyzed also the mtROS production, which was significantly increased in G+PA25 treatments as compared to G (Figure 6L; G+PA25: 1.3 ± 0.32 fold vs. G).

### 3.7. Mitochondrial Fission Inhibition Limits the Favorable Effects of PA 25 μM on pOBs Functionality

**Figure 7:**
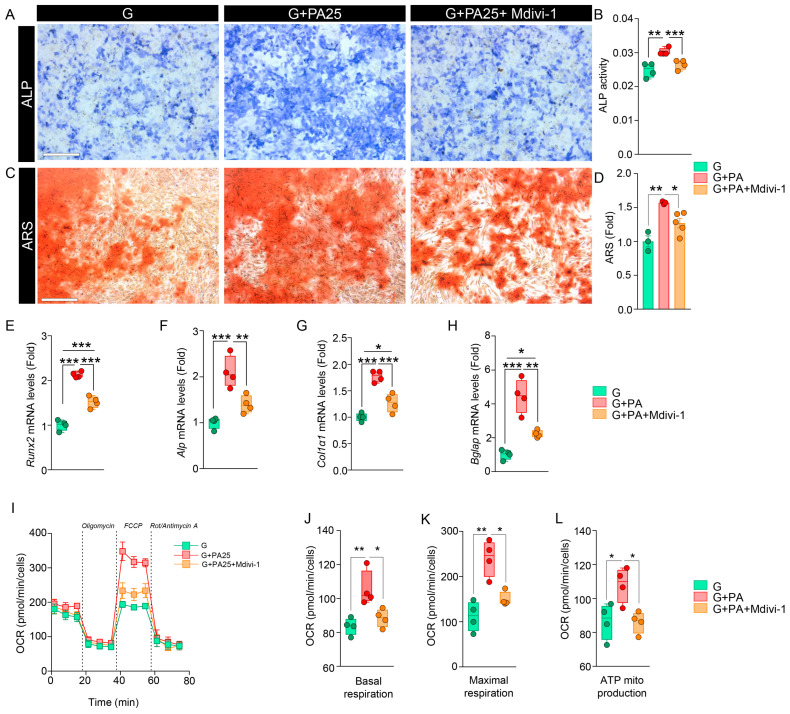
Mitochondrial fission inhibition effect on pOBs mineralization and metabolism in PA conditions. pOBs in osteogenic conditions treated with G or G+PA25 or treated with G+PA25+Mdivi-1 compound. (**A**) Representative images for ALP staining (Scale bar 400 μm) and (**B**) ALP enzyme activity (*n =* 4 per group). (**C**) Representative images for ARS staining (Scale bar 400 μm) and (**D**) relative ARS quantization (*n =* 3 for G, *n =* 3 for G+PA25, *n* = 5 for G+PA25+Mdivi-1). (**E–H**) qPCR analysis for osteogenic marker genes (*n =* 4 per group). Mitochondrial activity profile upon Mdivi-1 administration to G+PA25 in pOB. (**I**) Seahorse analysis on osteogenic-induced pOBs undergoing G, G+PA25, and G+PA25+Mdivi-1 treatments, and (**J**–**L**) evaluation of respiratory parameters expressed as OCR, pmol/min/cells (*n* = 4 per group). * *p* < 0.05, ** *p* < 0.01, *** *p* < 0.001.

To understand whether mitochondria fission is a key process by which mitochondria can sense the presence of glucose and non-cytotoxic PA levels and adapt pOBs energy metabolism, we treated the pOBs in the G+PA25 condition with the mitochondria division inhibitor compound, Mdivi-1 (G+PA25+Mdivi-1). We assessed pOBs functionality observing that when fission is inhibited, the G+PA25+Mdivi-1 incubation limited the increased osteogenesis induced by G+PA25 as compared to G alone. Specifically, ALP staining (Figure 7A) and activity (Figure 7B) were significantly reduced upon fission inhibition as compared to G+PA25 (ALP activity G: 0.0248 ± 0.002; G+PA: 0.03 ± 0.001; G+PA+Mdivi-1: 0.0265 ± 0.001). Accordingly, evaluation of ARS staining and its quantization (Figure 7C,D) showed a decreased mineralization in the G+PA25+Mdivi-1 treatment as compared to G+PA25 (ARS fold vs. G; G+PA25: 1.57 ± 0.02, G+PA+Mdivi-1: 1.2 ± 0.15). No differences were found between the control G condition and G+PA25+Mdivi-1 treatment, while, as expected, G+PA25 favored osteogenesis. Accordingly, gene expression analysis of osteogenic markers confirmed the trend of osteogenic-induced pOBs behavior previously observed, since the mRNA levels of the markers were reduced upon fission inhibitor treatment as compared to G+PA25 incubation, while not reaching the same levels as the G control (Figure 7E–H; *Runx2*, G+PA25: 2.13 ± 0.06, G+PA25+M-divi-1: 1.5 ± 0.12 fold vs. G; *Alp*, G+PA25: 2.1 ± 0.34, G+PA25+M-divi-1: 1.4 ± 0.19 fold vs. G; *Col1α1*, G+PA25: 1.7 ± 0.1, G+PA25+M-divi-1: 1.2 ± 0.17 fold vs. G; *Bglap*, G+PA25: 4.4 ± 1.01, G+PA25+M-divi-1: 2.2 ± 0.21 fold vs. G). As expected, we observed elevated expression levels of osteogenic genes in the G+PA25 group as compared to G. To corroborate these results, we performed OCR analysis in this experimental setting (Figure 7I) demonstrating that mitochondrial fission inhibition in the presence of both substrates by G+PA25+Mdivi-1 treatment significantly reduced respiratory parameters as compared to G+PA25 incubation (Figure 7J; basal respiration, G: 83.53 ± 4.9 OCR, G+PA25: 105.6 ± 10.41 OCR, G+PA25+Mdivi-1: 88.45 ± 5.32 OCR; Figure 7K; maximal respiration, G: 119 ± 32.71 OCR, G+PA25: 240.5 ± 39.91 OCR, G+PA25+Mdivi-1: 149.8 ± 15.81 OCR; Figure 7L; mitochondrial-linked ATP production, G: 86.6 ± 10.54 OCR, G+PA25: 108.1 ± 10.3 OCR, G+PA25+Mdivi-1: 85.98 ± 6.4 OCR). No differences were found between the control G condition and G+PA25+Mdivi-1 treatment, while as expected G+PA25 boosted mitochondrial respiration as compared to G alone.

## 4. Discussion

Our study showed that glucose and palmitate (PA)—the latter as the most abundant fatty acid (FA) in different tissues, in the circulation, and food—play a role in the control of osteoblasts (OBs) cellular physiology by regulating mitochondrial morphology and function. Several studies have demonstrated the lipotoxic effect of PA excess in several biological systems, including bone and OBs [14,15,16,18,19,34], highlighting the adverse effects of FA excess and unbalanced intake from the diet [14,34,35]. However, we have found that the addition of 25 μM PA in osteoblastic cells in vitro is not lipotoxic and that together with glucose, induces an increase in mitochondrial function favoring mineralization. 

Mitochondria play a pivotal role in orchestrating nutrient utilization by changing their morphology and adapting their activity in response to fuel availability [20,22,26]. PA-induced lipotoxicity has been associated with mitochondrial fission. [12,27,33,36,37]. In our studies, however, we have found that although the non-lipotoxic 25 μM PA dose induced mitochondrial fission process via activation of DRP1, this process is associated with enhanced mitochondrial respiration and mineralization. Interestingly, these data are in contrast with previous works reporting that during osteogenesis, human mesenchymal stromal (MSCs) show an interconnected elongated mitochondrial network, thus suggesting a mitochondrial fusion process [23,24]. This discrepancy could be due to the difference in cell models, the addition of PA in culture and the developmental stages that were used in our study compared to these previous reports, and the administration of PA during osteogenesis induction.

The relationship between mitochondrial dynamics and mitochondrial nutrient utilization is still to be clearly defined. Some studies have reported an association between FA metabolism and elongated mitochondria [38,39,40]. On the other hand, studies from our group have previously shown that in hypothalamic orexigenic neurons and microglial cells, FA utilization by the mitochondria is associated with mitochondrial fission [41,42,43]. Our current study seems to support such an association as we have found that in G+PA25-treated pOBs the addition of Mdivi-1, a DRP1 inhibitor, decreased mitochondrial respiration and negatively affected mineralization. However, as Mdivi-1 has been shown to reversibly inhibit Complex I independently from mitochondrial fission [44], future studies using transgenic mouse models of mitochondrial fission proteins in the osteoblast lineage cells are warranted to validate our observations. 

In our study, we have found that OBs adapt mitochondrial metabolism, function, and dynamics in order to maintain bioenergetic levels appropriate to cellular functions. The addition of a non-lipotoxic dose of PA to glucose increases the basal and maximal mitochondrial respiratory capacity, resulting in optimal ATP production that OBs mineralization. These data suggest that PA is an important nutrient whose utilization meets the energy demand of the OBs during mineralization. Indeed, our analysis of gene expression showed an elevation of genes involved in FA oxidation that provides approximately 40% to 80% of ATP, as demonstrated by several studies [8,45,46,47]. In support of this, it has been also demonstrated that lipid deprivation or loss of function of the Cpt1α enzyme in mouse skeletal stem cells impaired osteogenic differentiation, and loss of Cpt2 in mature OBs limited bone formation [9,48]. The reduction of mitochondrial respiratory rates observed in pOBs in G+PA25 condition with the mitochondria division inhibitor compound Mdivi-1 corroborates the hypothesis that, in this context, mitochondrial fission supports OBs functionality during osteogenesis.

## 5. Conclusions

In summary, the present in vitro study elucidates cellular and metabolic changes that occur in OBs favoring mineralization, in response to the presence of different energetic substrates, namely glucose and palmitate. By using the OBs cell line and primary osteoblastic cells we showed that the addition of a non-lipotoxic concentration of PA to glucose increased OBs functionality enhancing osteogenesis as compared to glucose treatment alone. This was associated with enhanced OBs mitochondrial function and altered mitochondrial dynamics supporting a fission process. Future in vivo studies evaluating bone physiology under different nutrient availability are warranted to better understand skeletal diseases associated with metabolic dysfunction such as obesity and type 2 diabetes, characterized by unbalanced circulating nutrient levels.

## Data Availability

Data is contained within the article or supplementary material.

## Supplementary Materials

The following supporting information can be downloaded at: https://www.mdpi.com/article/10.3390/nu15092222/s1, Supplementary Table S1, primers used for qPCR analysis; Supplementary Table S2, MTT test metabolic activity (Fold vs. T0) over time; Supplementary Figure S1. Effect of PA on MC3T3-E1 cell viability and metabolic activity by MTT Test over time. (A) MC3T3-E1 cells were treated for 96 h with Glucose 5.5 mM (G) plus different PA concentrations ranging from 12.5 μM to 800 μM. Cell counting is reported (*n* = 3 per group). * *p* < 0.05, ** *p* < 0.01 as compared to G treatment. (B) MTT assay metabolic activity relative fold to t0, over time (*n* = 6 per group). * *p* < 0.05, ** *p* < 0.01 as compared to G treatment at indicated time points. See Supplementary Table S2 for detailed analysis. Supplementary Figure S2. Effect of G+PA25 on mitochondrial mass and membrane potential in osteogenic-induced cells. (A) mtDNA copy number evaluation (*n* = 4 per group), (B) *Pgc1α* mRNA levels (Fold) (*n* = 4 per group) and (C) representative histograms relative to Mitotracker RED CMXRos fluorescence intensity reported in Figure 2I, in MC3T3-E1 cell line. (D) mtDNA copy number evaluation (*n* = 4 per group), (E) *Pgc1α* mRNA levels (Fold) (*n* = 4 per group) and (F) representative histograms relative to Mitotracker RED CMXRos fluorescence intensity reported in Figure 5I, in primary OBs. Supplementary Figure S3. Representative Western Blot original uncut image (relative to Figure 5L). Representative nitrocellulose membrane image for p-DRP1[Ser616], total DRP1 and β-actin determination in osteogenic-induced pOBs, derived from *n* = 3 animal samples (Wild type, WT C57Bl/6 mice) undergoing G and G+PA25 treatments in culture. The black rectangles identify the cut image reported in the main text in Figure 5L.
