# Supplementary material for: Nutrient-Dependent Mitochondrial Fission Enhances Osteoblast Function
Source: Nutrients. 2023 May 8;15(9):2222. doi: 10.3390/nu15092222 (PMC10181360; doi:10.3390/nu15092222)
Supplement: Supplementary file 1 [file nutrients-15-02222-s001.zip › Supplementary Figures and Legends_Menale et al..pdf]

Supplementary Figures and Legends

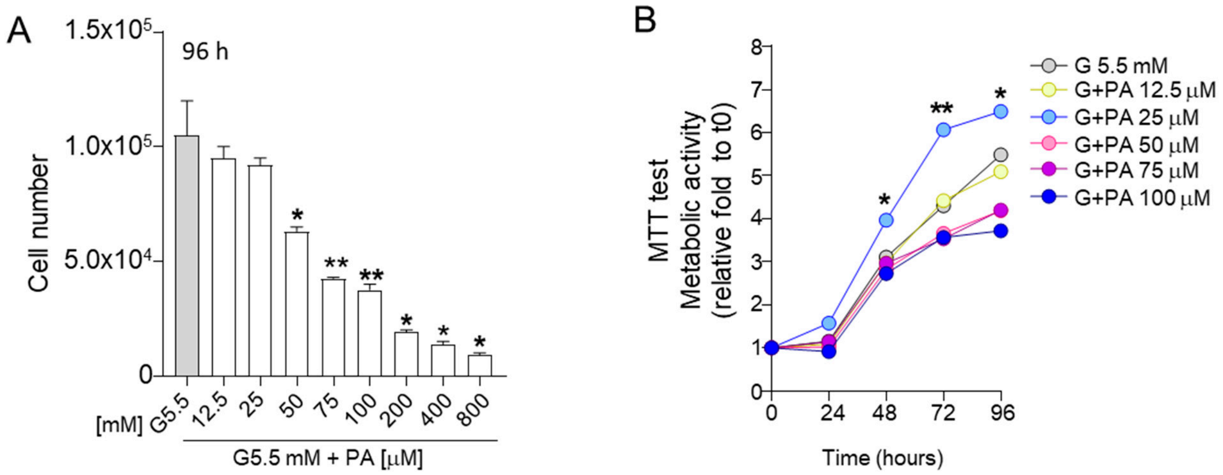

Supplementary Figure S1. Effect of PA on MC3T3-E1 cell viability and metabolic activity by MTT Test over time. (A) MC3T3-E1 cells were treated for 96 h with Glucose 5.5 mM (G) plus different PA concentrations ranging from 12.5 μM to 800 μM. Cell counting is reported ($n = 3$ per group). * $p < 0.05$, ** $p < 0.01$ as compared to G treatment. (B) MTT assay metabolic activity relative fold to t0, over time ($n = 6$ per group). * $p < 0.05$, ** $p < 0.01$ as compared to G treatment at indicated time points. See Supplementary Table S2 for detailed analysis.

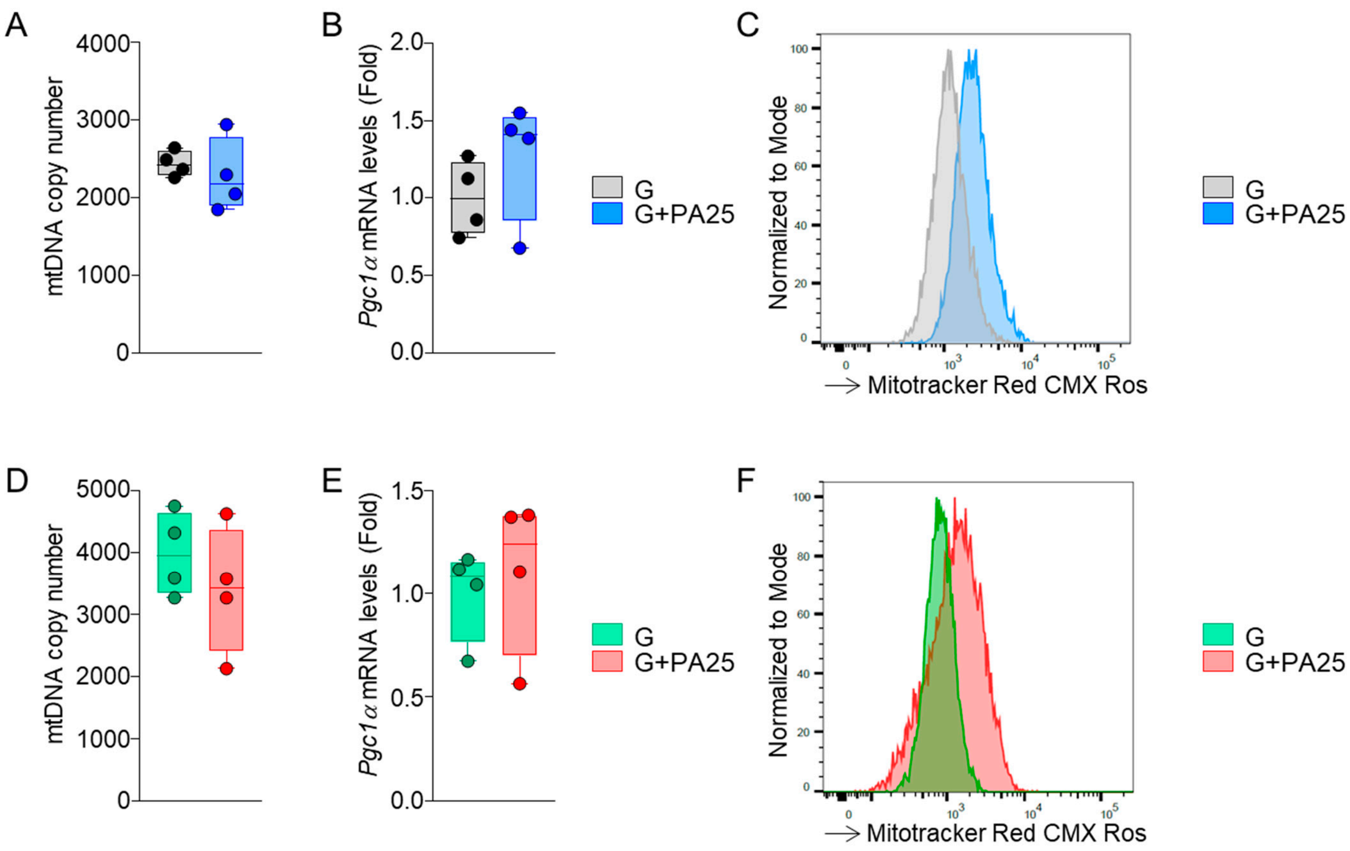

Supplementary Figure S2. Effect of G+PA25 on mitochondrial mass and membrane potential in osteogenic-induced cells. (A) mtDNA copy number evaluation (n = 4 per group), (B) *Pgc1α* mRNA levels (Fold) (n = 4 per group) and (C) representative histograms relative to Mitotracker RED CMXRos fluorescence intensity reported in Figure 2I, in MC3T3-E1 cell line. (D) mtDNA copy number evaluation (n = 4 per group), (E) *Pgc1α* mRNA levels (Fold) (n = 4 per group) and (F) representative histograms relative to Mitotracker RED CMXRos fluorescence intensity reported in Figure 5I, in primary OBs.

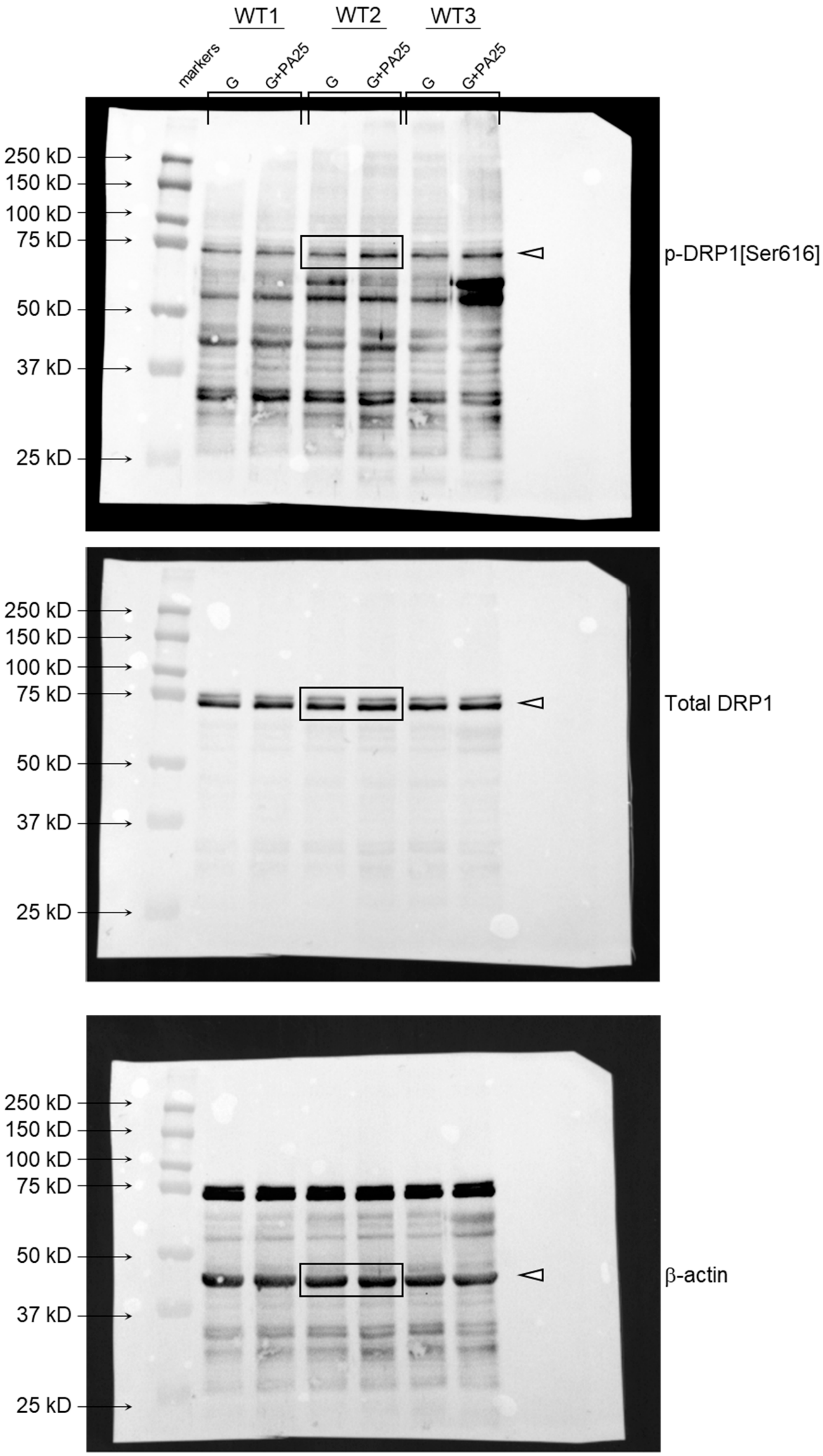

WT1
WT2
WT3
markers
G
G+PA25
G
G+PA25
G
G+PA25
250 kD
150 kD
100 kD
75 kD
50 kD
37 kD
25 kD
p-DRP1[Ser616]
250 kD
150 kD
100 kD
75 kD
50 kD
37 kD
25 kD
Total DRP1
250 kD
150 kD
100 kD
75 kD
50 kD
37 kD
25 kD
β-actin

Supplementary Figure S3. Representative Western Blot original uncut image (relative to Figure 5L). Representative nitrocellulose membrane image for p-DRP1[Ser616], total DRP1 and β-actin determination in osteogenic-induced pOBs, derived from *n* = 3 animal samples (Wild type, WT C57Bl/6 mice) undergoing G and G+PA25 treatments in culture. The black rectangles identify the cut image reported in the main text in Figure 5L.
