# Supplementary material for: Nutrient-Dependent Mitochondrial Fission Enhances Osteoblast Function
Source: Nutrients. 2023 May 8;15(9):2222. doi: 10.3390/nu15092222 (PMC10181360; doi:10.3390/nu15092222)
Supplement: Supplementary file 1 [file nutrients-15-02222-s001.zip › Supplementary Tables_1_2_Menale et al.pdf]

Supplementary Table S1. Primers used for qPCR analysis

Gene	Primer Fwd (5′-3′)	Primer Rev (5′-3′)
18s	GCTCTAGAATTACCACAGTTATCCAA	AAATCAGTTATGGTTCCTTTGGTC
Acadl	GACCTCTCTACTCACTTCTCCAG	TCTTTTCCTCGGAGCATGACA
Alp	CGGATCCTGACCAAAAACC	TCATGATGTCCGTGGTCAAT
Atp6	CAACCGTCTCCATTCTTTCC	TCATGTTCGTCCTTTTGGTG
Bglap	TGAGGACCATCTTTCTGCTCA	TGGACATGAAGGCTTTGTCA
Cd36	TTGAAAAGTCTCGGACATTGAG	TCAGATCCGAACACAGCGTA
Col1α1	CCGCTGGTCAAGATGGTC	CTCCAGCCTTTCCAGGTTCT
Cpt1α	TCTGCCATCTTGAGTGGTGA	GACTCCGCTCGCTCATTC
Cpt2	TCCCAATGCCGTTCTCAAAAT	CAGCACAGCATCGTACCCA
Mtco2	CCTCCACTCATGAGCAGTCC	GAATAACCCTGGTCGGTTTG
Nd1	GTGAGTGATAGGGTAGGTGCA	AACACTCCTCGTCCCCATTC
Pgc1α	TATGGAGTGACATAGAGTGTGCT	TCAAGAGCTGGTCCTTGTACC
RNase P	CTGACCACACGAGCTGGTAGAA	GCCTACACTGGAGTCGTGCTACT
Runx2	GCCCAGGCGTATTTCAGAT	TGCCTGGCTCTTCTTACTGAG

Supplementary Table S2. MTT test metabolic activity (Fold vs T0) over time.

	**MTT test Metabolic Activity (Fold±SD vs Time 0)**					
Time (hours)	*G (5.5 mM)*	*G+PA 12.5 μM*	*G+PA 25 μM*	*G+PA 50 μM*	*G+PA 75 μM*	*G+PA 100 μM*
24	1.16±0.12	1.10±0.09	1.57±0.10	1.02±0.10	1.14±0.10	0.91±0.10
48	3.10±1.05	2.30±0.99	3.97±1.66*	2.82±0.73	2.97±0.94	2.72±0.82
72	4.30±1.25	4.42±1.63	6.07±0.99**, ##	3.67±0.87	3.53±1.03	3.57±1.76
96	5.50±0.58	5.10±1.06	6.50±0.80*, ##	4.19±0.57*	4.20±0.79*	3.72±0.61*

*p<0.05, **p<0.01 vs G

##p<0.01 vs G+PA50 μM, G+PA75uM, G+PA100 μM
